# Embodied metaphors and interpersonal synchrony in the digital age: the case of remote working

**DOI:** 10.3389/fpsyg.2025.1648733

**Published:** 2025-08-01

**Authors:** Giulia Magni, Luana Amadini Genovese, Giuseppe Riva, Claudia Repetto

**Affiliations:** ^1^Department of Psychology, Catholic University of the Sacred Heart, Milan, Italy; ^2^Humane Technology Lab, Catholic University of the Sacred Heart, Milan, Italy; ^3^Applied Technology for Neuropsychology Lab, IRCCS Istituto Auxologico Italiano, Milan, Italy

**Keywords:** interpersonal synchrony, embodied cognition, technology-mediated communication, conceptual metaphor theory, face-to-face interaction, social neuroscience, remote working

## Abstract

This paper explores the impact of technology-mediated (TM) communication on interpersonal synchrony through the integrated lens of social neuroscience, embodied cognition, and Conceptual Metaphor Theory (CMT). It focuses particularly on the case of remote working, which exemplifies the challenges and adaptations required when social interactions shift from face-to-face (FTF) to digital environments. While FTF communication enables interpersonal synchrony through rich sensorimotor cues, such as gaze, posture, and gesture, TM communication often reduces or distorts these embodied signals. This disconnection undermines critical neurobiological processes like mirror neuron activation and impairs shared intentionality, emotional resonance, and trust. The paper highlights how embodied metaphors, especially CLOSENESS IS INTIMACY, are destabilized in digital contexts, making social engagement more cognitively demanding and less intuitive. Despite these limitations, humans show adaptability, developing compensatory behaviors and recalibrating expectations over time. However, remote working environments often lack the embodied coherence necessary to fully support collaborative creativity and social cohesion. The paper argues for the redesigning of digital platforms with immersive, multisensory interfaces, such as haptic feedback and spatialized audio, that better simulate the embodied dynamics of FTF interactions. It calls for future research employing hyperscanning and ecologically valid methods to understand how synchrony unfolds across digital modalities and how metaphorical coherence might be restored. Ultimately, by reengaging the body as a central substrate for cognition and communication, this work advocates for a more human-centered evolution of remote work technologies that sustains connection, trust, and collective performance.

## Introduction

Human cognition and identity are often shaped by social engagement. From early development through the lifespan, individuals’ well-being and sense of self are continuously shaped through interactions with others (e.g., eye contact and mirroring each other’s behavior during play between a baby and a parent supports both bonding and co-regulation of emotional states) (Kang et al., 2016). This relational orientation is not only psychological or cultural but deeply embodied, manifesting in the spontaneous coordination of movement, attention, and emotion that arises during interaction (Borghi et al., 2023). One of the most compelling expressions of this embodiment is interpersonal synchronization, which refers to the alignment of rhythms, gestures, physiological states, and even neural activity between people engaged in social exchange (Gallese, 2006). Interpersonal synchronization plays a critical role in fostering affiliative bonds, enhancing cooperation, and cultivating empathy (Hu et al., 2017; Leong et al., 2017). It has been observed in diverse contexts: lovers unconsciously mirroring each other’s postures, audiences matching performers’ heart rates during rituals, therapists and clients attuning breath and gaze (Konvalinka et al., 2011; Palumbo et al., 2017). Such synchronous dynamics support mutual understanding, regulate emotional states, and contribute to the co-construction of meaning. As demonstrated by research (Konvalinka et al., 2011; Fischer et al., 2013; Fujiwara et al., 2020), higher levels of synchronization are positively associated with stronger perceived relational bonds and trust. This phenomenon extends beyond observable behaviors. At the neural level, hyperscanning techniques have revealed correlations between interbrain synchronization and cooperative behaviors, shared comprehension, and joint attention (Dumas et al., 2010; Czeszumski et al., 2020). Physiologically, alignment in psychophysiological measures such as heart rate variability and electrodermal activity has been linked to emotional contagion, rapport, and group cohesion (Konvalinka et al., 2011; Golland et al., 2015). Synchrony is thus both an indicator and a facilitator of relational closeness, as evidenced by studies showing its role in enhancing group cooperation and interpersonal rapport (Hove and Risen, 2009; Wiltermuth and Heath, 2009; Gordon et al., 2020). More recently, scholars have begun to examine how interpersonal synchronization facilitates the emergence of collective mind representations, that is the experience of shared cognition and mutual awareness in group settings (Shteynberg et al., 2023). This involves more than shared knowledge; it reflects a recursive awareness where individuals not only know something but know that others know it, producing a deep sense of alignment. Moving in synchrony enhances this sense of joint agency and blurs the self-other boundary, promoting a collective “we-mode” of thinking (Pacherie, 2014; Miles et al., 2017).

The emergence of digital technologies has profoundly transformed the modalities of human interaction, raising critical questions about their effects on interpersonal synchrony, emotional attunement, and the neurobiological foundations of social cohesion. With the increasing prevalence of remote communication, researchers have turned to neuroscience to understand how mediated interaction differs from traditional face-to-face engagement (Riva et al., 2021). These issues are particularly salient in the case of remote working, which highlights the urgency of understanding how individuals adapt to digitally mediated forms of interaction. As technology-mediated practices like video conferencing, messaging, and online collaboration tools proliferate, it becomes increasingly important to investigate how these tools reshape interpersonal synchronization compared to traditional face-to-face contexts. While such technologies offer new forms of connectivity, they also limit spontaneous micro-coordination and nonverbal feedback, potentially undermining emotional resonance, trust, and group cohesion (Riva et al., 2024).

The present paper therefore draws on findings from social neuroscience and cognitive psychology, reading them through the lens of embodied cognition and Conceptual Metaphor Theory. Through the comparison of in presence and remote interactions, the aim is to identify how digital communication platforms alter the cognitive, affective, and physiological foundations of interpersonal synchrony. By integrating these frameworks, this perspective aims to elucidate the mechanisms that foster or hinder interpersonal synchrony and psychological closeness across physical and digital modes of interaction. The relevance of this research is especially evident in the context of remote working, where understanding the embodied and inferential dynamics of digital connection is essential for promoting effective collaboration and well-being in distributed teams. For this reason, this paper focuses on the case of remote working as a representative context in which embodied communication is challenged by technological mediation.

## Face-to-face communication and interpersonal synchronization

Face-to-Face (FTF) communication represents the most evolutionary embedded and ecologically valid form of social interaction. It is distinguished by a high number of non-verbal sensory cues (e.g., facial expressions, prosody, body posture, gestures), which enable a high level of informational and emotional transfer (Dikker et al., 2017; Fujihira et al., 2024). Indeed, FTF interactions allow individuals to convey a complex set of messages through bodily signals, such as facial expressions and gestures, facilitating deeper understanding and emotional resonance. These rich multimodal cues allow for spontaneous interpersonal synchrony, where individuals dynamically align their speech patterns, gestures, gaze, and even physiological states like heart rate and skin conductance (Golland et al., 2015). This synchrony facilitates a profound sense of co-presence and shared intentionality. Indeed, FTF communication is rooted in a shared physical space, which engages a series of neurobiological mechanisms essential for social bonding. Among these, there are mirror neurons, which enable individuals to intuitively simulate and understand the actions and emotions of others (Rizzolatti et al., 2001; Dumas et al., 2010), and von Economo neurons, which are involved in high-level social cognition and intentional attunement (Allman et al., 2011). These neural systems facilitate a rich, multi-sensory exchange that forms the basis of scaffolding in educational and mentoring contexts. Physical environments also engage hippocampal structures that underpin autobiographical memory and personal identity which anchor individuals in time and space, allowing them to integrate experiences into coherent self-narratives (Riva, 2023). This takes place through a phenomenon known as “placeness,” which addresses the unique emotional and cognitive significance that a place holds for an individual, such as the triggering of vivid memories when visiting a relevant place of our past (Relph, 1976). Shared physical contexts are a key element to generate interpersonal synchrony, which fosters shared intentionality and enhances group cohesion (Dikker et al., 2017). Another element that enables individuals to attune to each other’s states and intentions is rhythmic entrainment in speech and gesture, which is central to social meaning-making. Indeed, when people are physically co-present, they co-regulate their affective and attentional rhythms, often without conscious awareness (Gill, 2012). Specifically, research suggests that FTF communication favors the development of motor synchrony, which in turn fosters pro-social behaviors and promotes trust and empathy (Probolovski et al., 2022). FTF communication thus enables the activation of “we-mode” processes, which address a cognitive mode in which individuals act not merely as isolated agents but as part of a collective with shared goals and mutual commitment (Shteynberg et al., 2023). These embodied processes may indeed foster emotional regulation and support the development of trust.

Such benefits are especially evident in applied settings. In education, FTF activities enhance student connectedness and academic engagement (Chung-Do et al., 2013), while in healthcare, they strengthen patient trust and the sense of being cared for (Morgan et al., 2021). A growing body of research points to the workplace as another critical domain. Working in presence supports more collaborative and productive environments by providing richer non-verbal feedback and deeper engagement (Macchi and De Pisapia, 2024). It also enhances interpersonal synchronization, which is crucial for team coordination and shared understanding (Jiang et al., 2012; Yun, 2013).

In this light, the transition to remote working environments poses significant challenges. By reducing access to embodied cues and spontaneous micro-coordination, digitally mediated communication may hinder the emergence of synchrony and the cognitive-affective processes it supports. Understanding how remote work alters these mechanisms is thus crucial for designing digital tools and organizational practices that can approximate the benefits of FTF interaction and sustain trust, collaboration, and emotional resonance in virtual settings.

## Remote communication and the disruption of synchrony

The increase of digital and Technology-Mediated (TM) communication has naturally transformed interpersonal dynamics, facilitating more flexible and responsive interactions and representing a crucial solution to geographical and contextual limitations (Sanmas et al., 2024). Indeed, in situations characterized by restricted physical interactions, digital communication provides an alternative pathway to foster social connectedness (Nguyen et al., 2022). However, TM communication tends to alter the nature and quality of interpersonal synchrony. Research has shown that excessive reliance on digital devices for social interactions enhances feelings of isolation and disconnection and diminishes empathetic feelings (Bratina, 2023; Newson et al., 2024). Additionally, despite its potential for convenience and accessibility, this type of interaction typically lacks the spatial and sensory cues necessary to engage GPS neurons and facilitate autobiographical encoding. This leads to a phenomenon of “placelessness,” in which individuals experience environments as interchangeable and temporally fragmented, weakening the formation of coherent memories (Riva et al., 2021). Moreover, one of the most significant differences is the reduction or absence of non-verbal cues (e.g., body language, gaze direction, and physical proximity). This reduces sensory inputs, constrains embodied engagement, which may cause the diminished activation of mirror neurons and the impairment of intentional attunement (Riva et al., 2021). For instance, in videoconferencing, the visual field is often limited to the head and shoulders, excluding many body-based cues essential for embodied interaction: eye contact is often misaligned due to camera placement, and tactile cues are entirely absent (Riva et al., 2021; Clarin and Baluyos, 2022). Another aspect to take into consideration is the temporal disruption inherent in digital communication: latency, jitter, and asynchrony in audiovisual streams impede the fluid turn-taking and reciprocal responsiveness that characterize FTF dialogue (Fujihira et al., 2024). These temporal distortions disrupt the micro-coordination of speech and gesture, making it harder to establish the rhythmic predictability necessary for synchrony (Gill, 2012). Indeed, research shows that groups relying on videoconferencing appear to have lower group cohesion and performance compared to in-person teams (Macchi and De Pisapia, 2024; Sánchez Rodríguez et al., 2025). Overall, TM communication demands greater cognitive effort: in the absence of immediate embodied cues, individuals must consciously infer emotions and intentions, leading to increased cognitive load. This phenomenon, known as “Zoom fatigue,” is attributed to the sustained attention and interpretation required to process degraded or partial cues (Nesher Shoshan and Wehrt, 2022). A natural consequence of these aspects is the impairment of the development of “we-representations,” which are shared cognitive models that allow individuals to coordinate actions and intentions and ultimately lead to a diminished creativity, weakened group identification, and fragmented social bonds (Kourtis et al., 2019; Riva et al., 2021; Loehr, 2022).

Despite these challenges, humans demonstrate a notable capacity for adaptation in response to altered communicative environments. Indeed, individuals tend to develop new norms and compensatory rhythms in order to interact effectively in digital environments, and synchronous mutual adaptation can still occur, especially when participants are familiar with each other or highly motivated to connect (Gill, 2012). However, these forms of synchrony may not carry the same affiliative power as those arising naturally in FTF settings. In these contexts, the development of a shared intentionality and “we-mode” processes may indeed be hindered by the delay in the alignment of cues and the reduction of information. In the context of remote working, these insights are especially critical. While TM tools enhance task efficiency and flexibility, they can also impair key interpersonal processes, such as emotional attunement, collaborative creativity, and communication fluency, by undermining the synchrony that supports them (Riva et al., 2021). Understanding these dynamics is essential for designing digital work environments that preserve not just productivity, but also the relational quality and collective intelligence needed for effective teamwork.

## Embodied metaphors and digital closeness

An interesting approach for understanding the disruption of interpersonal synchrony in TM communication lies in the framework of Conceptual Metaphor Theory (CMT) and embodied cognition. According to CMT, abstract, social, and emotional experiences are understood through metaphorical mappings grounded in sensorimotor experience (Lakoff and Johnson, 1980). Among the primary metaphors that are most pervasive and relevant is CLOSENESS IS INTIMACY, which maps physical proximity onto emotional and cognitive connectedness (Lakoff and Johnson, 1980; Gallese and Lakoff, 2005). In FTF contexts, this metaphor is enacted and reinforced through embodied cues such as eye contact, bodily co-orientation, touch, and shared environmental references. These cues are not merely symbolic, but constitute perceptual simulations that activate sensorimotor systems and enable individuals to feel close in both spatial and psychological terms (Barsalou, 1999; Torres-Martínez, 2022). Echoing the assumption that the brain follows an inferential process, by continuously comparing incoming sensory input against predictive models derived from past experiences, the embodiment of metaphors serves as a prior experience (Clark, 2013). When sensorimotor cues align with metaphor-based expectations, such as feeling warmth when experiencing kindness or increased proximity to signal trust, prediction error is minimized, and the interaction is experienced as fluent and emotionally coherent (Magni et al., 2024). This process highlights the role of perceptual psychological mechanisms in shaping social understanding. For instance, shared intentionality is based on psychological mechanisms such as attentional focus and joint attention, which can modulate sensorimotor cues (i.e., eye gaze, body orientation) (Frischen et al., 2007). Similarly, proximity and movement cues influence affective and emotional abilities by addressing safety, warmth, and tension (Decety and Lamm, 2006). In turn, the perception of synchrony activates neural circuits associated with empathy and reward, reinforcing the affiliative value of coordinated interaction (Kokal et al., 2011).

In FTF settings, these embodied signals reliably support the metaphor of closeness, thus fostering interpersonal attunement. However, in TM communication, the degradation of audio-visual signals, latency, and the absence of bodily cues may impair the metaphorical scaffolding, leading to less intuitive and more effortful social engagement. The screen becomes a symbolic substitute for presence, but it does not replicate the full embodied experience of physically being near someone. Eye gaze is often misaligned, body language is truncated, and haptic feedback is missing (Riva et al., 2021). As a result, the embodied basis for the metaphor of closeness becomes destabilized, and with it, the intuitive sense of intimacy often associated with in-person interactions (Niedenthal et al., 2005; Landau et al., 2010). This disembodiment may fragment the coherence of social meaning-making and disrupt the inferential processes that underpin trust and rapport (Riva et al., 2024).

**Figure 1:**
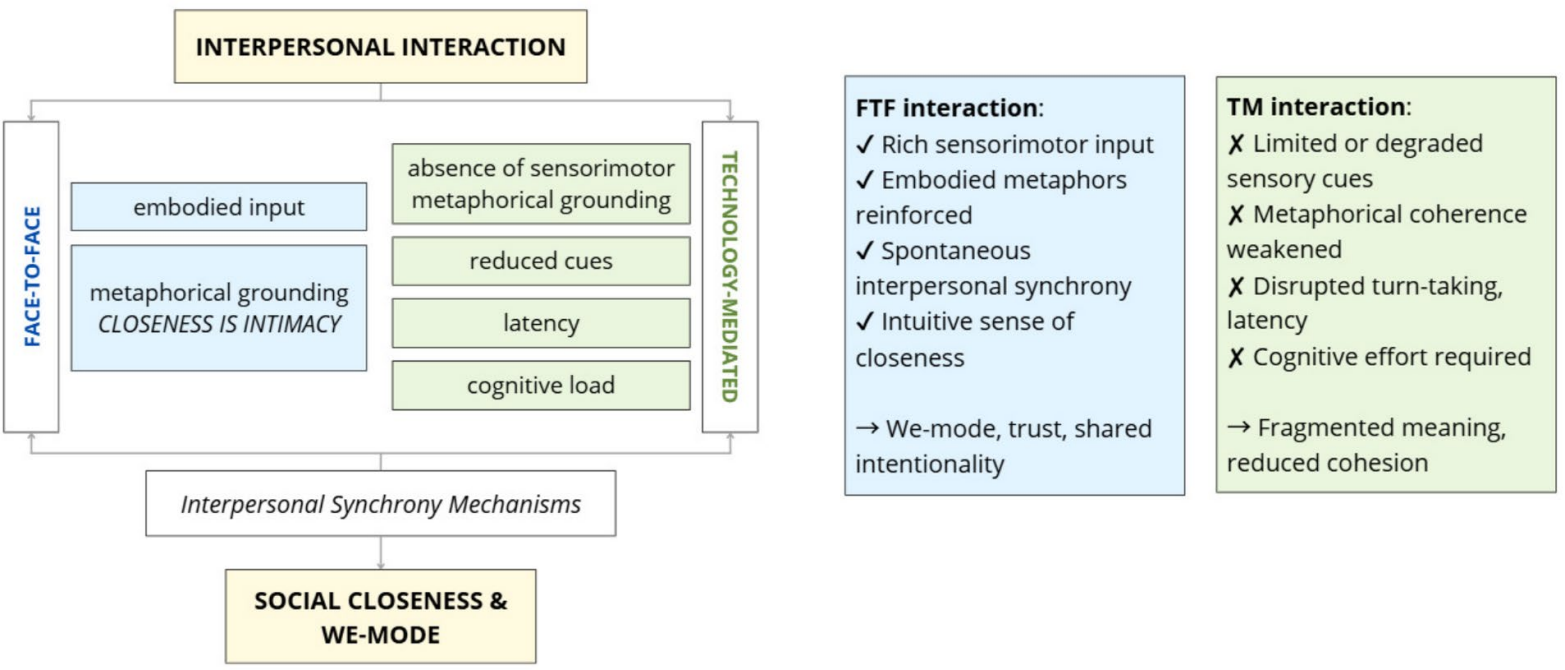
Conceptual model contrasting face-to-face (FTF; in blue) and technology-mediated (TM; in green) communication. The figure visually summarizes how FTF interactions foster interpersonal synchrony and we-mode processes via embodied cues and metaphorical grounding, while TM interactions disrupt this process, leading to weakened metaphorical coherence and diminished psychological intimacy.

However, metaphorical frameworks are not immutable. Through repeated exposure and adaptive learning, individuals may be able to recalibrate their predictive models to accommodate the constraints and affordances of digital environments. This is evident in the development of compensatory behaviors such as deliberate verbal affirmations, exaggerated facial expressions, or structured turn-taking cues (Gill, 2012). In remote working environments, where TM communication is the norm, this theoretical lens reveals how the loss of embodied metaphors may impair interpersonal closeness, team cohesion, and trust. However, it also highlights the human capacity for adaptation and the potential for designing digital interactions that foster new forms of meaningful connection grounded in alternative but effective metaphorical and sensorimotor frameworks (Figure 1).

## Re-engaging the body through embodied metaphors

The findings of this perspective underscore the importance of designing digital environments that support meaningful interaction, particularly in the context of remote working, where technology often substitutes for physical co-presence. Indeed, physical co-presence appears to foster interpersonal synchrony by grounding social connection in shared sensorimotor experience. Metaphors such as CLOSENESS IS INTIMACY are not mere linguistic constructs, but embodied scaffolds that enable individuals to feel more attuned with each other and socially present. However, remote work technologies often bypass or fragment these signals, resulting in disembodied exchanges that increase cognitive load and strain relational processes.

While tools such as video conferencing and messaging platforms offer efficiency and accessibility, they rarely replicate the full spectrum of embodied interaction. Virtual and augmented reality show promise in activating neural systems like GPS and mirror neurons, yet still face challenges in conveying the fluid, multisensory dynamics of in-person collaboration. To preserve these dynamics in digital settings, platforms should move beyond the visual-verbal interface and incorporate immersive, multisensory feedback. On this note, characteristics such as haptic signals and spatialized auditory stimuli become essential components for restoring the intuitive fluency and emotional resonance of FTF communication. This calls for a new generation of workplace technologies designed around predictive alignment and metaphoric resonance, i.e., interfaces that support natural rhythms of coordination and the embodied grounding of social meaning. By enhancing sensory richness and synchrony, such tools can mitigate the cognitive strain of remote communication and help sustain the affiliative power necessary for trust, creativity, and group flow. Indeed, by taking into account the dynamic interconnection between technological mediation, embodied metaphor, and interpersonal synchrony (further explored in Table 1), it is possible to develop more effective digital environments that support emotional attunement, relational trust, and cognitive coherence, which are necessary characteristics for meaningful social interactions in remote contexts.

**Table 1:** Conceptual relations between interpersonal synchronization, embodied metaphor, and technology mediation.

Concept	Definition	Connection to others concepts
Interpersonal synchronization	The alignment of actions, emotions, or physiological states.	Emerges from embodied interaction and is impaired when metaphorical coherence is absent due to degraded digital mediation.
Embodied metaphor	Abstract concepts (e.g., intimacy) grounded in bodily experiences (e.g., closeness).	Depends on sensorimotor cues (i.e., proximity, eye contact, …) to activate metaphorical frameworks.
Technology mediation	Use of digital platforms for social interaction.	Alters sensory inputs and embodied feedback.

The table defines each construct and its connection to the others within the context of remote social interaction.

In order to address the limitations of the current literature, such as its fragmentation, often lacking longitudinal and ecologically valid data that can capture the evolving nature of online sociality over time, future research must adopt integrative and valid methodologies. For instance, many studies focus on isolated variables, such as gaze alignment or latency, without fully addressing the complex interplay of cognitive, emotional, and sensorimotor processes in real-time interaction. Hyperscanning techniques, which enable simultaneous recording of neural activity in interacting individuals, could offer valuable insights into how synchrony unfolds across both physical and digital modalities as shown in literature (Liu et al., 2018; Nam et al., 2020; Sato and Sato, 2025). For example, to explore how neural and behavioral dynamics change in remote settings compared to FTF situations, researchers could combine hyperscanning with tasks carried out through a technology-mediated communication, such as collaborative problem-solving or decision-making activities. Moreover, experimental designs should incorporate immersive technologies such as virtual and augmented reality to assess whether these platforms can effectively simulate embodied presence and restore metaphorical coherence more effectively than traditional screen-based interfaces. Indeed, immersive technologies are characterized by a high sense of presence, which refers to the subjective experience of being physically present in a non-physical environment (Riva, 2008). The development of interfaces that support real-time mutual gaze, tactile feedback, and environmental embedding may prove critical for enhancing interpersonal attunement in digital settings. Examining the impact of these features on team trust, emotional regulation, and collaborative problem-solving will be vital for guiding the design of digital workspaces that promote both efficiency and human connection.

Ultimately, remote working environments demand not just technical solutions, but a deeper interdisciplinary understanding of how the body, brain, and technology co-evolve in shaping social experience. By reengaging the body as an active substrate for cognition and connection, future platforms can better replicate the synchrony and shared intentionality foundational to successful collaboration.

## Conclusion

The transition from FTF to digital communication represents more than a change in medium; it constitutes a transformation in the neurocognitive landscape of sociality. While digital tools offer unprecedented flexibility and accessibility, they often undervalue their impact on the embodied synchrony that underpins empathy, learning, and community cohesion. Understanding these dynamics through the lens of neuroscience can guide the development of more humane and effective digital platforms that support, instead of replacing, the intricate phenomenon of interpersonal attunement. In agreement with the Disembodied Disconnect Hypothesis (Riva et al., 2024), this perspective suggests that, although digital platforms offer novel ways of connection, they fail to engage the embodied, neurobiological processes essential for deep interpersonal understanding. Reintegrating bodily, rhythmic, and metaphorical dimensions into digital design may thus play an important role in sustaining meaningful human relationships in an increasingly virtual world. Within this approach, remote working becomes an illustrative case in which the relevance of digital embodiment and the implications of disrupted interpersonal synchrony are highlighted. It is therefore fundamental to address the need for digital infrastructures that can support the embodied dimensions of communication, thereby assisting the development of trust, collaboration, and social cohesion.

## Data Availability

The original contributions presented in the study are included in the article/supplementary material, further inquiries can be directed to the corresponding author.
